# Characterization of viral RNA splicing using whole-transcriptome datasets from host species

**DOI:** 10.1038/s41598-018-21190-7

**Published:** 2018-02-19

**Authors:** Chengran Zhou, Shanlin Liu, Wenhui Song, Shiqi Luo, Guanliang Meng, Chentao Yang, Hua Yang, Jinmin Ma, Liang Wang, Shan Gao, Jian Wang, Huanming Yang, Yun Zhao, Hui Wang, Xin Zhou

**Affiliations:** 1 Key Laboratory of Bio-Resources and Eco-Environment of Ministry of Education, College of Life Sciences, Sichuan University, Chengdu, 610065 China; 2 BGI-Shenzhen, Shenzhen, 518083 China; 3 China National GeneBank, BGI-Shenzhen, Shenzhen, 518120 China; 4 CAS Key Laboratory of Biomedical & Diagnostic Technology, CAS/Suzhou Institute of Biomedical Engineering and Technology, Suzhou, 215163 China; 5 James D. Watson Institute of Genome Sciences, Hangzhou, 310058 China; 6 The Institute of Biomedical Engineering, University of Oxford, Oxford, OX3 7DQ UK; 7 Beijing Advanced Innovation Center for Food Nutrition and Human Health, College of Plant Protection, China Agricultural University, Beijing, 100193 China; 8 National Engineering Research Center for Fruit and Vegetable Processing, China Agricultural University, Beijing, 100193 China; 9 Centre for GeoGenetics, Natural History Museum of Denmark, University of Copenhagen, Øster Voldgade 5-7, 1350 Copenhagen, Denmark

## Abstract

RNA alternative splicing (AS) is an important post-transcriptional mechanism enabling single genes to produce multiple proteins. It has been well demonstrated that viruses deploy host AS machinery for viral protein productions. However, knowledge on viral AS is limited to a few disease-causing viruses in model species. Here we report a novel approach to characterizing viral AS using whole transcriptome dataset from host species. Two insect transcriptomes (*Acheta domesticus* and *Planococcus citri*) generated in the 1,000 Insect Transcriptome Evolution (1KITE) project were used as a proof of concept using the new pipeline. Two closely related densoviruses (*Acheta domesticus* densovirus, AdDNV, and *Planococcus citri* densovirus, PcDNV, *Ambidensovirus*, *Densovirinae*, *Parvoviridae*) were detected and analyzed for AS patterns. The results suggested that although the two viruses shared major AS features, dramatic AS divergences were observed. Detailed analysis of the splicing junctions showed clusters of AS events occurred in two regions of the virus genome, demonstrating that transcriptome analysis could gain valuable insights into viral splicing. When applied to large-scale transcriptomics projects with diverse taxonomic sampling, our new method is expected to rapidly expand our knowledge on RNA splicing mechanisms for a wide range of viruses.

## Introduction

As increasing number of next-generation sequencing (NGS) datasets are being produced from various-omics initiatives, transcriptome sequencing of flora and fauna for a specific developmental stage/condition gains its popularity in biological research. Transcriptomics is implemented in discoveries of novel transcripts, SNPs, gene splicing and fusion, in determination of gene structure, function and regulation, and in quantification of expression levels^1^. It has already contributed a great deal of understanding to the mechanisms of functional elements, genes and transcripts^2,3^.

RNA splicing plays a vital role in genetics by increasing mRNA and protein diversities and by regulating gene expressions, providing an important link between genetic variation and disease^4–7^. Alternative splicing (AS) is one of the major mechanisms in increasing the diversity of proteins translated from a limited number of genes in metazoans^8,9^. The spliceosome complex, composed of at least 170 proteins and several small nuclear RNAs (snRNAs), is the key structure responsible for splicing in eukaryotes^10^. The complex defines exons/introns in transcribed RNAs by three major sequence elements: the 5′ splice site (donor site), the 3′ splice site (acceptor site), and the branch point^4,11^. When compared with annotated genome sequences, transcriptome sequencing could identify gene splicing isoforms and expression patterns associated with biological functions^12,13^.

Deep sequencing experiments often detect gene expressions not only for the focal taxon but also for pathogens infecting the host^14,15^. This feature has been known as dual-sequencing^16^. Viruses and endogenous viral elements occur in most organisms, including fungi, plants and animals^17–19^. Viruses also play pivotal roles in ecological systems^20^. In recent years, many novel viral infections have been discovered using NGS^21–24^. Several methods and tools have been developed for virus detection^25,26^, viral gene expression and host adaptation^15^ using NGS datasets. Among these, viral sequences are often assembled *de novo* using all reads or those not matched to the host genome^27–30^.

Transcriptome datasets are one of the most preponderant resources, in which host and viral components are both recorded^12,31^. Combined with genomic data, transcriptome sequencing has been used to detect known and novel disease-causing viruses^27,32,33^, to observe viral mutagenesis and recombination^26,34–36^, and to understand virus-host interactions^26,37^. RNA splicing plays important roles in viral replication and virus-host interactions^38^. Viral gene expression and RNA splicing are exclusively dependent of the host genomics machinery^39,40^, therefore the whole transcriptome datasets generated from host species (containing mRNA from both host and infecting virus) are good resources for revealing viral RNA splicing characteristics. There are more than 4,000 viral genomes publically accessible, even though more viruses are yet to be described^24,41^.

Two transcriptomes obtained from the 1000 Insect Transcriptome Evolution project (1KITE, www.1kite.org)^42^ were analyzed in this study: house cricket (*Acheta domesticus* (Linnaeus), Orthoptera, Gryllidae) and citrus mealybug (*Planococcus citri* (Risso), Hemiptera, Pseudococcidae). Previous studies suggest these insects often carry closely related viruses - *Acheta domesticus* densovirus (AdDNV) and *Planococcus citri* densovirus (PcDNV), respectively^43,44^. Densoviruses (family *Parvoviridae*) are widely distributed among arthropods^19,45^ with linear single-stranded DNA genomes of approximately 5,000-nucleotides, including two major gene cassettes encoding viral nonstructural (NS or Rep) and structural proteins (VP or CP)^43,45^. Densoviruses employ RNA AS to produce the nonstructural protein 1 (NS1) endonuclease using a rolling-hairpin mechanism to regulate replication^46,47^. Two NS transcripts and one VP transcript were detected in AdDNV and four splicing junctions were reported^48^.

**Figure 1:**
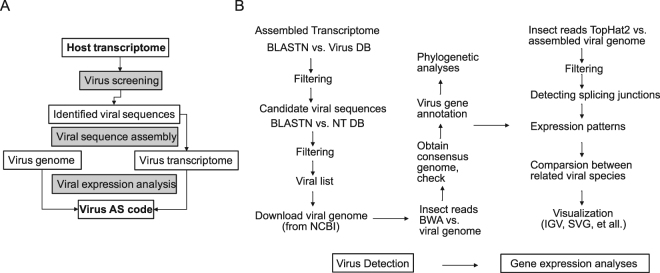
Analysis framework. (**A**) Analysis framework; (**B**) Detailed analytical pipeline. Virus detection and viral expression analyses: this pipeline was designed to detect and obtain viral sequences from transcriptome datasets; all in house Perl scripts used in the pipeline are available on web (https://github.com/linzhi2013/Virusfishing).

In the present study, we completed a NGS-based informatics pipeline to: 1) detect virus from the whole assembled transcriptome; 2) obtain viral genome sequence by calling consensus sequence from virus reads; 3) characterize virus AS codes and reveal gene expression patterns of the virus (Fig. 1). Using the 1KITE transcriptome, we characterized the splicing patterns in AdDNV and PcDNV, demonstrating both shared and unique splicing patterns in closely related viruses.

## Results

### Data description

The transcriptomes of *A*. *domesticus* and *P*. *citri* were generated by the 1KITE project^42^. In brief, total RNA was isolated from one *A*. *domesticus* juvenile female, collected in Hamburg, Germany, February 2013, and ca. 150 *P*. *citri* individuals, collected in Brandenburg, Germany, November 2011, respectively, using TRIzol (Invitrogen, Grand Island, NY, USA). The mRNA was isolated using the Dynabeads mRNA Purification Kit following the manufacturer’s protocol (Invitrogen, Grand Island, NY, USA). The mRNA extracts were treated with RNA fragmentation reagent (Ambion, Austin, Texas, US). Two cDNA libraries were constructed using SuperScript™II Reverse Transcriptase (Invitrogen, Grand Island, NY, USA), random N6 primer (IDT), RNase H (Invitrogen, Grand Island, NY, USA) and DNA polymerase I (New England BioLabs, Ipswich, MA, USA). The cDNA libraries were sequenced with the 150 bp paired-end strategy and 250 bp insert-size using Illumina’s HiSeq. 2000 platform (Illumina, San Diego, CA) at BGI-Shenzhen. The resulting sequences were subject to Illumina’s read quality control pipeline. 2.36 Gb (16,898,600 reads with 150 bp read size) and 2.89 Gb (20,670,410 reads with 150 bp read size) of high quality sequence data were obtained for *A*. *domesticus* (NCBI Accession No: PRJNA286330) and *P*. *citri* (NCBI Accession No: PRJNA219593, published by Misof *et al*.^42^), respectively.

### Viral sequence detection and calling

Both viral and Nt databases applied in our study were downloaded from the GenBank (accessed in Nov. 2014). The virus database contains 1,561,606 viral sequences (2.2 Gb) including nearly 5,000 complete viral genomes and the Nt database contains 29,059,038 sequences reaching a data size of 84.0 Gb.

**Figure 2:**
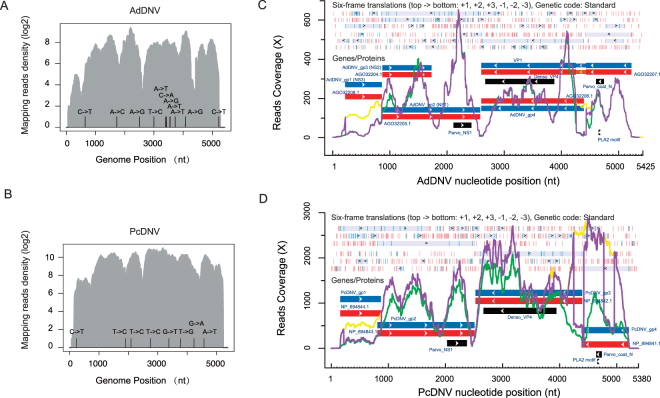
Genome coverage and annotations of AdDNV and PcDNV. Genome coverage of (**A**) AdDNV and (**B**) PcDNV. Log2 scale of read density was based on genomic sequences of AdDNV and PcDNV. Vertical bars highlight mutation sites against the reference sequences. Annotations of (**C**) AdDNV and (**D**) PcDNV. Coverage (Y-axis) of each nucleotide position (X-axis) was plotted for AdDNV_1KITE and PcDNV_1KITE. Six reading-frames and previously described genes were represented using information provided by NCBI, including: start/stop codons (short blue/red vertical bars), transcription directions (black arrows, from top to bottom: forward reading frames +1, +2, +3 and reverse reading frames −1, −2, −3), ORFs (solid gray boxes). Virus genes (blue boxes), proteins (red boxes) and conserved motifs (black boxes) were represented according to the NCBI annotations. BWA mapping profiles (green lines), TopHat2 mapping profiles (purple lines), TopHat2 gap mapping profiles (yellow lines, the number of both splicing and non-splicing reads, correspond to splicing junctions) were represented according to the mapping results. AdDNV introns reported in existing studies include: In (nt 223 to 855), Ia (nt 4403 to 4758), Ib (nt 4403 to 4544) and II (nt 4260 to 4434).

After virus detection and false positive (sequence matched to non-viral subjects) removal, the assembled transcriptome sequences had best matches with AdDNV in *A*. *domesticus* and with PcDNV in *P*. *citri* (Fig. 1B, Supplementary Table S1 and Supplementary Text S1). Near full-length consensus genomes of AdDNV (NCBI Accession No: KX145610) and PcDNV (NCBI Accession No: KX145609) were called based on the templates of viral reference genome, with 5,259 bases (96.94% of the AdDNV reference KF015278.1 with 6,084 mapped reads) and 5,220 bases (97.03% of the PcDNV reference NC004289 with 33,604 mapped reads), respectively. The missing regions were located at 5′ and 3′ ends for both viruses and were replaced by Ns for the following analysis. Eleven single nucleotide variants (SNVs) were detected for AdDNV and eight SNVs for PcDNV (Fig. 2A and B, Supplementary Table S2 and Supplementary Data file S1). None of these SNVs were located in splicing sites while some SNVs resulted in nonsynonymous mutations in ORF translations, i.e., six out of eleven SNVs in AdDNV and five out of eight in PcDNV (Supplementary Data file S1 Column N). Phylograms based on genome sequences and deduced proteins confirmed that AdDNV and PcDNV were closely related species within *Ambidensovirus* (Supplementary Fig. S1 and Supplementary Text S1)^43,49–57^.

### Expression of viral genes in transcriptome

Compared to the unspliced aligner BWA^58^ (Supplementary Table S2), TopHat2^59,60^ obtained much greater depth coverages (Supplementary Table S3 and Supplementary Data file S2) due to successful alignments of fragmented (gapped) transcripts onto reference genomes. AdDNV_1KITE obtained 6,090 mapped reads (998,487 bases) with an average depth coverage of 189×, and a highest coverage of 659× on a single site in the NS region (Fig. 2C). The PcDNV_1KITE consensus sequence had 40,101 reads mapped against the genome at an average coverage of 1,325× and a highest coverage of 2,785× on a single site in the VP region (Fig. 2D). To examine whether the difference in sequencing depth may affect the viral gene expression patterns, we carried out downsampling analyses using proportions (1/10 and 1/20) of the PcDNV dataset (details provided in the Validation Section).

Several conserved protein domains play important roles in DNA replication, gene expression, infection and transfection in DNVs^46,61,62^. Conserved domains, including Parvo_NS1 located in NS proteins, Pavo_coat_N, Denso_VP4 and phospholipase A2 (PLA2) motif located in VP proteins appeared to be highly expressed in both transcriptomes^63,64^ (Fig. 2 and Supplementary Text S1). In the meantime, the unevenness of coverage also suggested possibilities of novel transcripts or other special characters in particular regions that were prone or hard to be enriched during the library construction.

### Splicing profiles and introns of two viruses

Seven AS patterns are commonly reported in many species: exon skipping (SE), mutually exclusive exons (MXE), intron retention (IR), alternative 3′ sites (A3SS), alternative 5′ sites (A5SS), alternative first exon (AFE) and alternative last exon (ALE)^8,65,66^. In our findings, five introns were detected in AdDNV_1KITE that involved three AS patterns, i.e., A5SS, A3SS and IR (Table 1, Supplementary Table S4). All five AdDNV introns belonged to the canonical intron GT-AG type^67^.

**Table 1:** Detected introns of AdDNV and PcDNV.

Species	ID	reads supports	location	direction	Length (base)	Intron Type	Note
AdDNV	AdDNV_I1	88	223..855	+	633	GT-AG	A5SS, RI
	AdDNV_I2	3	431..855	+	425	GT-AG	A5SS, RI
	AdDNV_I3	7	4245..4533	−	289	GT-AG	A3SS
	AdDNV_I4	135	4260..4434	−	175	GT-AG	RI
	AdDNV_I5	18	4403..4533	−	131	GT-AG	A3SS
PcDNV	PcDNV_I1	43	217..879	+	663	GT-AG	A5SS, RI
	PcDNV_I2	275	221..879	+	659	GT-AG	A5SS, RI
	PcDNV_I3	1	287..879	+	593	GT-AG	A5SS, RI
	PcDNV_I4	20	304..879	+	576	GT-AG	A5SS, RI
	PcDNV_I5	10	689..879	+	191	GT-AG	A5SS, RI
	PcDNV_I6	3	710..879	+	170	GT-AG	A5SS, RI
	PcDNV_I7	4	770..879	+	110	GT-AG	A5SS, RI
	PcDNV_I8	1	1188..1299	+	112	GT-AG	RI
	PcDNV_I9	3	2721..2820	−	100	GT-AG	A3SS, RI
	PcDNV_I10	2	2740..2820	−	81	GT-AG	A3SS, RI
	PcDNV_I11	1	3721..3906	−	186	GT-AG	A3SS, RI
	PcDNV_I12	291	3824..3897	−	74	GT-AG	A5SS, RI
	PcDNV_I13	44	3824..3906	−	83	GT-AG	A5SS,A3SS, RI
	PcDNV_I14	3	4198..4480	−	283	GT-AG	A3SS
	PcDNV_I15	1	4249..4340	−	92	GT-AG	A5SS, RI
	PcDNV_I16	1994	4249..4423	−	175	GT-AG	A5SS, A3SS
	PcDNV_I17	7	4249..4480	−	232	GT-AG	A5SS, A3SS, SE
	PcDNV_I18	3	4281..4423	−	143	GT-AG	A3SS
	PcDNV_I19	1	4341..4423	−	83	GT-AG	A3SS
	PcDNV_I20	429	4403..4480	−	78	GT-AG	A3SS, RI, SE
	PcDNV_I21	2	4775..4852	−	78	GT-AG	A5SS, RI
	PcDNV_I22	6	4775..4898	−	124	GT-AG	A5SS, RI
	PcDNV_I23	1699	4775..4958	−	184	GT-AG	A5SS, RI
	PcDNV_MI1	3	4249..4340;4403..4480	−	92;78	GT-AG;GT-AG	SE

**Figure 3:**
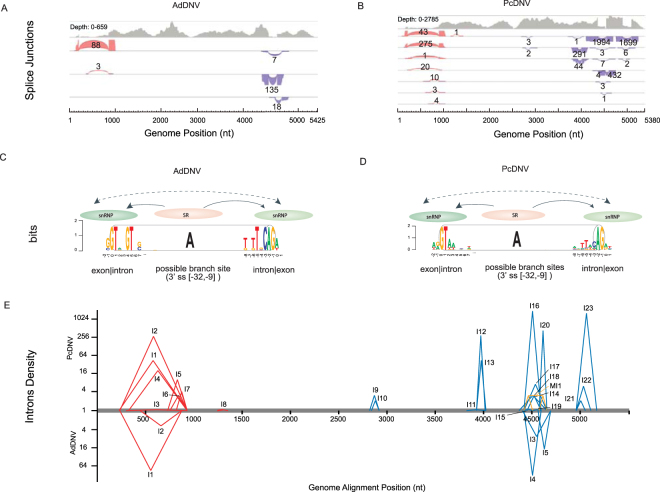
Splicing profiles of AdDNV and PcDNV. (**A**) Detected splicing junctions of AdDNV_1KITE. (**B**) Detected splicing junction models of PcDNV_1KITE: Solid gray areas represented the TopHat2 mapping profiles and each color-coded block represented a splicing junction. Red and purple blocks were forward and reverse junctions, respectively. The edge of each block represented the coverage of supporting reads and the length of a block represented the location of a splicing event. The number near each block was the coverage of supporting reads. The middle bridge showed the intron region from the splicing event. The block thickness represented frequency (the number of supporting reads) of the intron. Splice site compositions for donor sites, branch sites and acceptor sites of all GT-AG type introns in AdDNV_1KITE (Panel C) and PcDNV_1KITE (Panel D) were displayed using WebLogo. The overall height of each stack indicated the sequence conservation at that position, measured in bits. Proteins mediating the GT-AG splicing were labelled as snRNP (small nuclear ribonucleoproteins) and SR (splicing regulatory proteins). (**E**) Log2 scale of reads density of introns in the genome alignment: The Y-axis showed the expression levels (the number of reads) of intron related splicing events. Introns with forward junctions (red labels, at NS region) and reverse junctions (blue labels, at VP region) of PcDNV_1KITE (top half) and AdDNV_1KITE (bottom half) were shown in the genome alignment. Multiple splicing events (orange labels) were also displayed.

**Figure 4:**
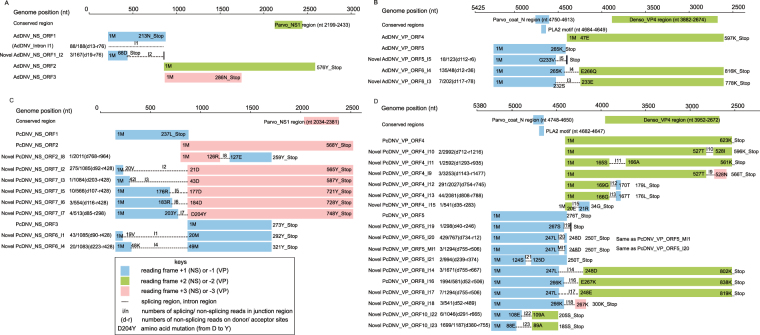
Inferred viral gene products. Viral gene products were annotated according to viral genome positions. The NS genes were represented in forward direction (Panels A and C) and the VP genes were represented in reverse direction (Panels B and D). For the splicing products, numbers of the detected splicing reads/non-splicing reads (over the intron) were listed next to the gene ID (covering the donor and receptor junctions). Numbers of the non-splicing reads of the donor (d) and receptor (r) sites were also labeled. Positions of start codons, stop codons and amino acids at splicing junctions were shown in the reading frames of forward (NS) and reverse (VP) polarities.

Detected splicing junction regions matched to previously reported AdDNV introns In (nt 223 to 855), Ib (nt 4403 to 4544) and II (nt 4260 to 4434), which were determined by Sanger sequencing of RT-PCR products^48^. Two A5SS introns, AdDNV_I1 (Table 1, nt 223 to 855, identical to the previously described In) and AdDNV_I2 (Table 1, nt 431 to 855) occurred in NS transcriptions. Two A3SS introns (Table 1, AdDNV_I3, nt 4245 to 4533; and AdDNV_I5, nt 4403 to 4533, the same as previously described Ib) and an IR intron (Table 1, AdDNV_I4, nt 4260 to 4434, previously described as II) occurred in the VP transcriptions^48^. In short, two novel introns (AdDNV_I2, _I3) and three known introns (AdDNV_I1, _I4, _I5) were detected while a previously reported intron (Ia, nt 4403 to 4758) was not detected in this research. Reads supported splicing junction models were showed in Fig. 3A by Integrative Genomics Viewer (IGV)^68^. Nine long open reading frames (ORFs) ranging from 207 to 2,451 nt were detected in the AdDNV_1KITE genome (Table 2, Fig. 4), six of which had been previously validated by experiments^48^. Five of the ORFs did not require splicing whereas the other four were splicing products.

**Table 2:** Viral gene products and their expression levels.

Species	Name	Regions	Involved Splicing sites	Nucleotide length (nt)	Effective length	FPKM (RSEM)	Relative expression level (%)	Product characters	NR Best hit overview	Putative Gene products
AdDNV	AdDNV_NS_ORF1	225..866	none	642	403	18362.89	5.59	Known	NS3 (AdDNV)	nonstructural protein NS3
	AdDNV_NS_ORF1_I2	join(225..430, 856)	AdDNV_I1 with depth 3	207	1	0	0.00	Truncation (C-terminal)	NS3 (AdDNV)	nonstructural protein
	AdDNV_NS_ORF2	856..2586	none	1731	1492	328484.48	100.00	Known	NS1 (AdDNV)	nonstructural protein NS1 with rolling-circle replication motif, walker/NTPase motif and Parvo_NS1 region
	AdDNV_NS_ORF3	875..1735	none	861	622	0	0.00	Known	NS2 (AdDNV)	nonstructural protein NS2
	AdDNV_VP_ORF4	c(2605..4398)	none	1794	1555	65660.96	19.99	Known	NS2 (AdDNV)	structural protein with Denso_VP4 region
	AdDNV_VP_ORF5	c(4424..5230)	none	807	568	28846.15	8.78	Known	putative structural protein (AdDNV_gp5)	structural protein 2 with Parvo_coat_N and PLA2 motif regions
	AdDNV_VP_ORF5_I5	c(join(4398..4402, 4534..5230))	AdDNV_I5 with depth 18	702	463	8673.58	2.64	Truncation (C-terminal); non-synonymous Mutation (G233V)	putative structural protein (AdDNV_gp5)	structural protein with Parvo_coat_N and PLA2 motif regions
	AdDNV_VP_ORF6_I4	c(join(2605..4259, 4435..5230))	AdDNV_I4 with depth 135	2451	2212	164855.44	50.19	Known; ORF shift (C-terminal); Non-synonymous mutation (E266Q)	structural protein VP1 (AdDNV)	structural protein VP1 with PLA2 motif, Parvo_coat_N and Denso_VP4 regions
	AdDNV_VP_ORF6_I3	c(join(2605..4244, 4534..5230))	AdDNV_I3 with depth 7	2337	2098	7443.55	2.27	Deletion	structural protein VP1 (AdDNV)	structural protein with PLA2 motif, Parvo_coat_N and Denso_VP4 regions
PcDNV	PcDNV_NS_ORF1	160..873	none	714	469	17344.87	6.42	Known	NS3 (PcDNV)	nonstructural protein NS3
	PcDNV_NS_ORF2	810..2516	none	1707	1462	15339.25	5.68	Known	NS1 (PcDNV)	nonstructural protein NS1 with Parvo_NS1 region
	PcDNV_NS_ORF2_I8	join(810..1187, 1300..1701)	PcDNV_I8 with depth 1	780	535	59.63	0.02	ORF shift (C-terminal)	NS1 (PcDNV)	nonstructural protein
	PcDNV_NS_ORF3	880..1701	none	822	577	0	0.00	Novel	Hypothetical protein MPH 12776	nonstructural protein NS2
	PcDNV_NS_ORF6_I1	join(160..216, 880..1701)	PcDNV_I1 with depth 43	879	634	18452.28	6.83	ORF shift (C-terminal)	putative nonstructural protein (PcDNV, PcdVgp4)	nonstructural protein
	PcDNV_NS_ORF6_I4	join(160..303, 880..1701)	PcDNV_I4; splicing reads depth: 20	966	721	814.32	0.30	ORF shift (C-terminal)	putative nonstructural protein (PcDNV, PcdVgp4)	nonstructural protein
	PcDNV_NS_ORF7_I2	join(160..220, 880..2516)	PcDNV_I2 with depth 275	1698	1453	182580.84	67.63	Combination	NS1 (PcDNV)	nonstructural protein with Parvo_NS1 region
	PcDNV_NS_ORF7_I3	join(160..286, 880..2516)	PcDNV_I3 with depth 1	1764	1519	108.68	0.04	Combination	NS1 (PcDNV)	nonstructural protein with Parvo_NS1 region
	PcDNV_NS_ORF7_I5	join(160..688, 880..2516)	PcDNV_I5 with depth 10	2166	1921	729.65	0.27	Combination	NS1 (PcDNV)	nonstructural protein with Parvo_NS1 region
	PcDNV_NS_ORF7_I6	join(160..709, 880..2516)	PcDNV_I6 with depth 3	2187	1942	218.03	0.08	Combination	NS1 (PcDNV)	nonstructural protein with Parvo_NS1 region
	PcDNV_NS_ORF7_I7	join(160..769, 880..2516)	PcDNV_I7 with depth 4	2247	2002	177.05	0.07	Combination; Mutation (D204Y)	NS1 (PcDNV)	nonstructural protein with Parvo_NS1 region
	PcDNV_VP_ORF4	c(2531..4402)	none	1872	1627	107736.5	39.91	Known	putative structural protein (PcDNV, PcdVgp2)	structural protein with Denso_VP4 region
	PcDNV_VP_ORF4_I9	c(join(2602..2720, 2821..4402))	PcDNV_I9 with depth 3	1701	1456	212.52	0.08	ORF shift (C-terminal)	putative structural protein (PcDNV, PcdVgp2)	structural protein
PcDNV	PcDNV_VP_ORF4_I10	c(join(2531..2739, 2821..4402))	PcDNV_I10 with depth 2	1791	1546	0	0.00	Deletion	putative structural protein (PcDNV, PcdVgp2)	structural protein
	PcDNV_VP_ORF4_I11	c(join(2531..3720, 3907..4402))	PcDNV_I11 with depth 1	1686	1441	3190.9	1.18	Deletion	putative structural protein (PcDNV, PcdVgp2)	structural protein
	PcDNV_VP_ORF4_I12	c(join(3789..3823, 3898..4402))	PcDNV_I12 with depth 291	540	295	319.37	0.12	ORF shift (C-terminal)	putative structural protein (PcDNV, PcdVgp2)	structural protein
	PcDNV_VP_ORF4_I13	c(join(3789..3823, 3907..4402))	PcDNV_I13 with depth 44	531	286	210.38	0.08	ORF shift (C-terminal)	putative structural protein (PcDNV, PcdVgp2)	structural protein
	PcDNV_VP_ORF4_I15	c(join(4206..4248, 4341..4402))	PcDNV_I15 with depth 1	105	0	0	0.00	ORF shift (C-terminal)	putative structural protein (PcDNV, PcdVgp2)	structural protein
	PcDNV_VP_ORF5	c(4392..5222)	none	831	586	0	0.00	Known	putative structural protein (PcDNV, PcdVgp1)	structural protein with PLA2 motif and Parvo_coat_N regions
	PcDNV_VP_ORF5_I19	c(join(4336..4340, 4424..5222))	PcDNV_I19 with depth 1	804	559	0	0.00	Truncation (C-terminal)	putative structural protein (PcDNV, PcdVgp1)	structural protein with PLA2 motif and Parvo_coat_N regions
	PcDNV_VP_ORF5_I20(MI1)	c(join(4392..4402, 4481..5222))	PcDNV_I20 with depth 429; or PcDNV_MI2 with depth 3	753	508	1659.65	0.61	Deletion	putative structural protein (PcDNV, PcdVgp1)	structural protein with PLA2 motif and Parvo_coat_N regions
	PcDNV_VP_ORF5_I21	c(join(4392..4774, 4853..5222))	PcDNV_I21 with depth 2	753	508	119.93	0.04	Deletion	putative structural protein (PcDNV, PcdVgp1)	structural protein with PLA2 motif and Parvo_coat_N regions
	PcDNV_VP_ORF8_I14	c(join(2531..4197, 4481..5222))	PcDNV_I14 with depth 3	2409	2164	614.74	0.23	Combination	putative structural protein (PcDNV, PcdVgp2)	structural protein with PLA2 motif, Parvo_coat_N and Denso_VP4 regions
	PcDNV_VP_ORF8_I16	c(join(2531..4248, 4424..5222))	PcDNV_I16 with depth 1994	2517	2272	185726.27	68.79	Combination; Mutation (E267K)	putative structural protein (PcDNV, PcdVgp2)	structural protein with PLA2 motif, Parvo_coat_N and Denso_VP4 regions
	PcDNV_VP_ORF8_I17	c(join(2531..4248, 4481..5222))	PcDNV_I17 with depth 7	2460	2215	166.07	0.06	Combination	putative structural protein (PcDNV, PcdVgp2)	structural protein with PLA2 motif, Parvo_coat_N and Denso_VP4 regions
	PcDNV_VP_ORF9_I18	c(join(4177..4280, 4424..5222))	PcDNV_I18 with depth 3	903	658	103.55	0.04	ORF shift (C-terminal)	putative structural protein (PcDNV, PcdVgp1)	structural protein with PLA2 motif and Parvo_coat_N regions
	PcDNV_VP_ORF10_I22	c(join(4481..4774, 4899..5222))	PcDNV_I22 with depth 6	618	373	203.45	0.08	ORF shift (C-terminal)	putative structural protein (PcDNV, PcdVgp1)	structural protein
	PcDNV_VP_ORF10_I23	c(join(4481..4774, 4959..5222))	PcDNV_I23 with depth 1699	558	313	269981.32	100.00	ORF shift (C-terminal)	putative structural protein (PcDNV, PcdVgp1)	structural protein

Note:

c: abbreviation of complement.

ORF shift: the open reading frame had a novel reading frame pattern produced by splicing, which was different from previously reported genes.

Relative expression level: the FPKM value of one gene divided by the FPKM value of the highest expressed gene of the same virus.

Parvo_NS1 region: AdDNV_1KITE: nt 2119 to 2433, reading frame + 1; PcDNV_1KITE: nt 2034 to 2381, reading frame + 3.

Denso_VP4 region: AdDNV_1KITE: nt 3882 to 2674, reading frame -2; PcDNV_1KITE: nt 3952 to 2672, reading frame −1.

Parvo coat N region: AdDNV_1KITE: nt 4750 to 4613, reading frame -1; PcDNV_1KITE: nt 4748 to 4650, reading frame −3.

PLA2 motif region: AdDNV_1KITE: nt 4684 to 4649, reading frame -1; PcDNV_1KITE: nt 4682 to 4647, reading frame −3.

Introns of AdDNV_I1, _I4 and _I5 were supported by 88, 135 and 18 reads, respectively (Table 1, Supplementary Table S4). These detected introns were congruent with experimental results from a previous study^48^, demonstrating that the NGS approach and bioinformatics applied in our study were reliable. The missing of a previously reported AdDNV (Intron Ia, nt 4403 to 4758) might be caused by inter-individual differences. In addition, two novel introns (AdDNV_I2 and AdDNV_I3) were detected at low read numbers (3 and 7, respectively, Table 1). Three out of four introns were validated using RT-PCR (details provided in the Validation Section). AdDNV_I2 was confirmed demonstrating that NGS was sensitive in detecting introns at very low expression levels.

The splicing junctions and AS pattern of PcDNV are reported in the present study for the first time (Fig. 3B). The A5SS and A3SS introns occurred in PcDNV with high frequencies. Seven out of eight NS introns belonged to A5SS and all shared the same 3′ site at nt 879 (Fig. 3E). In the VP encoding region, four splicing islands were detected and all of them contained either A5SS or A3SS or both modes (Fig. 3E and Table 1). Five in 23 introns had only a single read support suggesting limited function if there were any. All introns belonged to canonical GT-AG introns^69^. Twenty-eight ORFs ranging from 531 to 2,517 nt were detected in the PcDNV genome (Table 2, Fig. 4). Five of the ORFs, as in AdDNV, did not require RNA splicing whereas the others (23 out of 28) were splicing products.

Different from AdDNV, PcDNV lacked the major AdDNV_I1 intron in the NS region. Instead, PcDNV displayed a set of seven A5SS introns with a shared receptor junction at nt 879. With different donor positions, the PcDNV NS introns produced a set of novel proteins composing NS ORF1 and NS ORF2 (Fig. 4C). The dominant NS splicing was PcDNV_NS_ORF7_I2. Other seven splicing events in NS region produced more additional isoforms of the ORF1-ORF2 protein and ORF1-ORF3 protein (Fig. 4C). 17 out of the 28 ORFs were located in VP region. The most common VP splicing in PcDNV was PcDNV_VP_ORF8_I16 (Fig. 4D). Like the previously described AdDNV_VP_ORF6_I4, PcDNV_VP_ORF8_I16 eliminated the stop codon of PcDNV_VP_ORF5 and joined PcDNV_VP_ORF4 (homologue of AdDNV-VP2) reading frame. Downsampling tests of PcDNV showed similar results (details in Validation Section), demonstrating that the differences observed between the two viruses were not solely caused by sequencing depth. More detailed descriptions of gene products of the two viruses could be found in Supplementary Text S1.

### Gene expression features of AdDNV and PcDNV

#### Gene expressions of AdDNV_1KITE

By calculating the ratio of the number of splicing and non-splicing reads spanning the exon-intron regions and the FPKM/TPM values of the viral ORFs, expression patterns were compared between AdDNV and PcDNV. Both RSEM^70^ (Table 2) and Kallisto^71^ (Supplementary Table S6) produced similar profiles. Junctions with high splicing/non-splicing ratios also produced transcripts with high FPKM/TPM values. In AdDNV, the ratio of AdDNV_I4 was the highest in AdDNV (Supplementary Table S4) and AdDNV_NS_ORF2 (Fig. 4A) had the highest FPKM indicating that the encoded AdDNV_NS1 was the most abundantly expressed protein (Table 2) while AdDNV_VP_ORF6_I4 was the mostly expressed VP isoform (VP1) (Fig. 4B).

#### Gene expressions of PcDNV_1KITE

PcDNV_I16 had the highest splicing/non-splicing ratio in PcDNV, followed by PcDNV_I23 (Supplementary Table S4). Based on the FPKM values, PcDNV_NS_ORF7_I2, PcDNV_NS_ORF6_I1 and PcDNV_NS_ORF1 were the most abundantly expressed PcDNV_NS proteins (Fig. 4C, Table 2). Although PcDNV_VP_ORF8_I16 encoded a VP protein similar to AdDNV_VP_ORF6_I4 (AdDNV_VP1), its FPKM value was second to that of PcDNV_VP_ORF10_I23 which encoded a novel protein without any conserved *Ambiensovirus* VP motifs^48,57,72^ (Fig. 4B and D, Table 2). The PcDNV_NS proteins had smaller FPKM values than the PcDNV_VP proteins, suggesting that PcDNV_NS proteins were expressed less abundantly than the PcDNV_VP proteins (Table 2). This was different to the situation found in AdDNV.

#### Expression patterns of viral genes

Expression patterns of the viral transcripts were significantly different between AdDNV and PcDNV on two levels, i.e., transcript isoforms and expression abundance (Fig. 4 and Table 2). Most of the spliced transcripts had low FPKM values or even effectively zero count (for those with small effective lengths), suggesting that these rare splicing products were unlikely to be responsible for any fundamental viral function^48,57,72^. On the other hand, as splicing products dominated in both NS and VP of AdDNV and PcDNV, RNA splicing played essential roles in these two densoviruses. Differential splicing resulted in remarkable divergence of the viral transcriptome (Figs 3 and 4).

The two viruses were phylogenetically closely related (Supplementary Fig. S1) therefore were expected to adopt similar gene expression strategies including AS^48,52^. Indeed, the splicing junctions were located in both NS and VP regions in AdDNV and PcDNV (Fig. 3A and B) and IR, A5SS and A3SS modes of alternative splicing were also detected for PcDNV in this study. The majority of the PcDNV GT-AG splicing occurred at a region similar to the AdDNV splicing hotspot (Fig. 3E), i.e., PcDNV_I16 (covered by 1,994 reads) and AdDNV_I4 (covered by 135 reads) were both positioned at the genome alignment region from nt 4,596 to 4,418. This structural consistency indicated that this splicing event was conserved across species and likely played an important role in densoviruses (Fig. 3E, Table 1, Supplementary Table S4).

On the other hand, novel splicing events, including the canonical GT-AG introns with SE modes (Table 1), were discovered for PcDNV. There were more splicing events in PcDNV than in AdDNV, even if the same level of sequencing depth was tested in each sample. The question whether or not such a difference may be caused by the number of host individuals sampled remains open. In general, the PcDNV consensus was less restricted than that of AdDNV, particularly at both donor (+4G and +5T) and acceptor positions (−3C and −5T) (Fig. 3C and D, Supplementary Fig. S3, Supplementary Text S1). All five AdDNV introns belonged to the canonical intron GT-AG type^67^. It is worth noting that the consensus sequences of these GT-AG introns (nG|GTAnGTnG for donor and TnTTGCAG|An for acceptor, Fig. 3C) were different to the corresponding consensus sequences of GT-AG introns in PcDNV (AG|GTAAnnnn for donor and AnTTACAG|AT for acceptor with all junctions, AG|GTAAnnnn for donor and AATTACAG|AT for acceptor when junctions of very low frequencies (<3 reads) were excluded, Fig. 3D, Supplementary Fig. S3). Both viral consensus sequences had differences with the conserved consensus of U2 type GT-AG introns (AG|GTRAGT for donor and YYTTYYYYYYNCAG|G for acceptor) or U12 type introns (|RTATCCTTT for donor and TTCCTTRAY for branch sites) (Fig. 3C and D, Supplementary Fig. S3, Supplementary Text S1)^69,73^.

### Validating splicing products using bioinformatics and experimental methods

#### Downsampling of the PcDNV dataset

To examine potential effects of sequencing depth on resultant splicing patterns, we randomly subsampled 1/10 and 1/20 of the PcDNV dataset and performed identical analyses on these subsamples. The 1/10 subsample had 2,067,041 reads in total, with 8,428 reads mapped to PcDNV after filtering (Supplementary Table S3). 12 out of 23 splicing junctions recovered from the full dataset were detected, including all major junctions (read depth > 50) and some (4 out of 14) minor junctions with low read depths (read depth < 10, Supplementary Tables S3 and S4). Similarly, the 1/20 subsample had 1,034,316 reads in total with 4,806 reads mapped to PcDNV after filtering. Although the mapped viral reads in the 1/20 subsample of PcDNV was fewer than that from AdDNV (6,090), more splicing junctions were detected (9 as oppose to 5 in AdDNV) (Supplementary Tables S3 and S4). These results showed that increases of sequencing depth enhanced the number of rare splicing junctions but had no impact on the detection of major splicing junctions.

#### Splicing results from RNA-seq aligner STAR

We also analyzed splicing patterns using STAR^74^. In AdDNV, the five introns detected by TopHat2 were also supported by STAR (Supplementary Tables S3 and S4). In PcDNV, STAR detected 28 introns (23 by TopHat2), including 23 GT-AG introns that were also detected by TopHat2 and five additional rare splicing junctions supported by single read (Supplementary Table S4).

#### Junctions validation using RT-PCR method

RNA extracts remaining from the production of the two transcriptomes were used for RT-PCR validations (Supplementary Table S7). Primers were designed based on ORF sequences revealed by viral genome assemblies and annotation from this study to amplify regions of the splicing junctions. Several ORFs could share the same primers (Table 3, Supplementary Table S8, Supplementary Fig. 4). As expected, RT-PCR amplified ORFs with the most abundant junctions or highest expression levels sharing the same primers. AdDNV_I2, _I4 and _I5 (read depths of 3, 135 and 18, respectively) and PcDNV_I1, _I2, _I16 and _I23 (read depths of 43, 275, 1994 and 1699, respectively) were validated by Sanger sequencing of the RT-PCR products^75^. However, some rare junctions with low coverage depths were not confirmed by this method. The results showed that deep sequencing was more sensitive in detecting rare junctions than the RT-PCR based approach. On the other hand, it is also possible that splicing junctions supported by a single read might be caused by sequencing and/or mapping errors.

**Table 3:** RT-PCR summary.

Number	Name	Length	Designed PCR product length	PCR gel results	Splicing detected by Snger	Detected Junctions	Primer
1	AdDNV_NS_ORF1	642	631	600~700 bp	√		AdDNV_ORF1_F1, _R1
2	AdDNV_NS_ORF1_I2	207	207	near 200 bp	√	I2	AdDNV_ORF1_F1, AdDNV_ORF1_I2_R1
3	AdDNV_NS_ORF2	1731	1704	near 2 kb	√		AdDNV_ORF2_F1, _R1
4	AdDNV_NS_ORF3	861	842	700–1 kb	√		AdDNV_ORF3_F1, _R1
5	AdDNV_NS_ORF4	1794	1794	near 2 kb	√		AdDNV_ORF4_F1, _R1
6	AdDNV_NS_ORF5	807	807	700–1 kb	√		AdDNV_ORF5_F1, _R1
7	AdDNV_NS_ORF5_I5	702	702	600–1 kb	√	I5	AdDNV_ORF5_F1, AdDNV_ORF5_I5_R1
8	Ad_DNV_NS_ORF6_I3	2451	2337	2kb–3kb	not detected	I4	AdDNV_ORF6_F1, AdDNV_ORF4_R1
	Ad_DNV_NS_ORF6_I4	2337			√		
9	PcDNV_NS_ORF6_I1	879	966	near 1 kb	√	I1	PcDNV_ORF6_F1, _R1
	PcDNV_NS_ORF6_I4	966			not detected		
10	PcDNV_NS_ORF7_I2	1698	1660	near 2 kb	√	I2	PcDNV_ORF7_F1, _R1
	PcDNV_NS_ORF7_I3	1764			not detected		
	PcDNV_NS_ORF7_I5	2166			not detected		
	PcDNV_NS_ORF7_I6	2187			not detected		
	PcDNV_NS_ORF7_I7	2247			not detected		
11	PcDNV_VP_ORF4	1872	1872	near 2 kb	√		PcDNV_ORF4_F1, _R1
	PcDNV_VP_ORF4_I10	1791			not detected		
	PcDNV_VP_ORF4_I11	1686			not detected		
12	PcDNV_VP_ORF8_I14	2409	2517	2kb–3kb	not detected	I16	PcDNV_ORF8_F1, PcDNV_ORF4_R1
	PcDNV_VP_ORF8_I16	2517			√		
	PcDNV_VP_ORF8_I17	2460			not detected		
13	PcDNV_VP_ORF10_I22	618	558	500–900 bp	not detected	I23	PcDNV_ORF10_F1, _R1
	PcDNV_VP_ORF10_I23	558			√		

## Discussion

Virus infections are common in most eukaryotic organisms^30^. Deep sequencing of transcriptomes coupled with bioinformatics pipeline developed in the present study can readily detect transcripts of the target organism (host) as well as those of the pathogens. In our pipeline, we firstly screened the assembled sequences (scaffolds) for potential viral sequences against a customized viral database instead of the full Nt database. The resulting viral candidate sequences were then screened against the Nt database to remove false positives (sequences matched to non-viral subjects). The outcome of this two-steps virus screening is the same as directly using the assembled sequences to screen against the Nt database. However, virus screening using two large datasets (assembled sequences and Nt database) consumes much greater computer resource. Therefore, our pipeline for virus detection improved computational efficiency without compromising on accuracy. Given that a wide range of transcriptomes for non-model organisms have been *de novo* assembled by a series of large-scale transcriptome projects, including the 1KITE^42,76,77^, this practice provides an effective pathway to characterize virus diversity across various lineages of life.

In addition, our study demonstrated that transcriptome sequencing was an effective and accurate approach to improve the understandings of gene catalogues, expression levels, and RNA splicing patterns for pathogens. Viral gene expression profiles can be identified from the transcriptomes (Figs 3 and 4). Transcriptomic analysis in a phylogenetic context can help to elucidate functional conservativeness and novelty of expressed genes. For instance, phylogenetically closely related viruses, AdDNV and PcDNV, exhibited both conserved transcriptions and lineage-specific profiles. Common VP1 isoforms were abundantly transcribed using highly conserved splicing events (AdDNV_VP_ORF6_I4 and PcDNV_VP_ORF6_I16, Fig. 4), suggesting that these proteins were essential for the virus survival. On the other hand, differences in structural and non-structural proteins inferred from viral transcripts were found between the two viruses, including novel sequence divergences and distinctive variations in expression ratios and levels.

The diverse splicing mechanisms in PcDNV seemed to have directly led to more varieties of gene splicings than that in AdDNV (Fig. 3E, Table 1, Supplementary Tables S4 and S5) and these novel introns inturn produced new protein isoforms. The number of pooled individual specimens in the 1KITE project was justified according to the body weight of insects to provide sufficient RNA for transcriptome sequencing, and it could cause limitations in sample variation and inadequacies in detecting of inter-individual differences in the current study. Variations introduced by sequencing depth (1,325× versus 189×), number of pooled individual specimens (150 versus 1), temporal variation in gene expressions and inter-individual difference may have also contributed to some of the unique patterns observed in PcDNV, especially some of the rare introns. In future research, analyses on more virus-host pairs with controlled individual numbers could help to clarify the effects of different factors. RT-PCR based validations verified the majority of major novel splicing junctions, but most junctions with low read support could not be confirmed. Therefore, the possibility of artificial errors cannot be completely ruled out for expressed junctions at very low read coverage. Empirical validation of rare splicing junctions is a challenging task. Although the Sanger sequencing of RT-PCR products has successfully verified a number of major and minor junctions, the conventional method is far not as sensitive as NGS. Therefore, interpretation of rare slicing junctions should be cautious until additional technologies are developed for validation and functional testing.

Nevertheless, transcriptome sequencing of host and co-expressed pathogen creates a unique opportunity to examine host-virus association. To our knowledge, our report on AS consensus sequences were the first description for PcDNV and AdDNV. Viruses rely on the host machinery for RNA biology and can co-evolve with the host splicing^78^. It is likely that the observed divergence of viral RNA splicing patterns (Fig. 3) were influenced by both host and viral factors. Additional pipelines for analyzing the host splicing patterns may help us to understand the virus-host interaction and co-evolution in the future.

## Methods

### Virus detection

Raw reads were assembled using SOAPdenovo-Trans^79^ with the following settings: “-K 31 –i 20 -e 3 –M 3 –L 100”^42^. We then searched for matches against a customized virus database (described below) using the Basic Local Alignment Search Tool (BLASTn, version 2.2.26)^80^, including 6 steps (Fig. 1B).

#### Customized virus database

All known viruses in non-redundant nucleotide (Nt) databases and their corresponding taxonomic identity were downloaded from the GenBank (November 2014) and served as the virus reference database, which was much smaller than the complete Nt database but more dedicated to viruses. This modification improved screening efficiency and alleviated computational demand.

#### Virus search

Assembled transcriptome scaffolds were searched for sequence homology against the customized virus reference database using a local BLASTN algorithm (e value < 1e-5). Only query sequences with a match-length ≥200 nt and identity ≥90% were retained as candidate viral sequences for further analyses. Overlapping candidate viral sequences were merged by combining the BLASTN results using an in house Perl script (available at https://github.com/linzhi2013/Virusfishing) with improved procedures in query selection (merged candidate viral sequences versus whole scaffolds or sequencing reads)^81^.

#### Removal of false positives

Candidate viral sequences were compared to the complete Nt database using BLASTN aiming to identify false positives. Sequences with higher scores to non-virus subjects were deemed as false positives and subsequently removed from downstream analyses.

#### Viral consensus genome calling

Virus genomes were achieved by calling consensus sequences. Candidate reference genomes (based on sequence homology from previous steps) were chosen from viral database to aid the following calling of relevant virus genome. Burrows-Wheeler Aligner (BWA, version 0.7.10)^58^ was applied to align all raw reads of the transcriptome onto virus reference genomes with default parameters. After removal of PCR redundancy using SAMtools (If multiple read pairs have identical external coordinates, only the pair with highest mapping quality was retained) and reads with more than two mismatches, consensus viral sequences were obtained using SAMtools (version 0.1.19)^82^, with ambiguous sites substituted by the base of highest allele frequency and with missing sites replaced by Ns.

#### Genome coverage and annotation

ReSeqTools (version 0.23)^83^ and self-developed Perl scripts were used to calculate the genome coverage and read depth. Viral gene information was downloaded from the GenBank. All translation start site and translation termination site at both directions were annotated based on prediction of amino acid sequences.

#### Phylogram construction

The virus phylogram was inferred using both genome sequences and protein sequences of the assembled viruses, reference viruses and other nine related viruses from the same family downloaded from GenBank. MUSCLE^84^ was applied to conduct multiple sequence alignments (MSA) and MEGA5 was used for tree construction using the maximum likelihood method^85^ and Neighbour-Joining method^86^ with 1000 bootstraps (MEGA5 software)^87^.

### Gene expression analyses

Gene expressions were analysed in five steps:

#### Detection of alternative splicing

TopHat2 (version tophat-2.0.7)^59,88^ was applied to indentify RNA splicing patterns based on junction signals. Alternative splicing events were identified accroding to gene splicing patterns. All transcriptome reads were mapped to reference viral genome assemblies and host database using paramters “-r 10 -i 50 -I 2000–library-type fr-unstranded –G” according to the TopHat2 manual, respectively. TopHat2 aligns reads that are spanning across gaps onto a reference more efficiently than the unspliced aligners, such as BWA and Bowtie^60,89^. RNA mapping characteristics were obtained from high-quality BAM files with unique mapping reads after removing of PCR redundancy and reads with more than two mismatches.

#### Determination of AS junctions, introns and gene expression patterns

Splicing junction locations and counts were obtained from spliced sequences that might contain one or two junctions per read. Non-canonical splicing sites with low number of supporting (n < 3) reads were filtered out from further analysis. And the putative branch site regions were identified in introns by searching regions relative to the 3′ splicing site^67^, using an in house Perl script. Sequence logos of all detected junctions and junctions with coverage depth ≥ 3 were generated by WebLogo^90^ and consensus sequences were generated by the tool *cons* from EMBOSS^91^, respectively. Introns and splicing patterns were identified according to the letters on donor and acceptor sites of the splice junctions^69,73,92,93^. The splicing level of an intron was recorded as read counts mapped to a junction, similar to the method by MATS^94^ in calculating the exon inclusion level. Integrative Genomics Viewer (IGV)^68,95^ was used to show the putative viral AS and intron models.

#### Annotation of viral open reading frames

Firstly, the putative coding regions of the viral genome were identified using an in house Perl script. Putative expression products with amino acid length > 30 aa were defined according to the open reading frames (ORFs) from the unspliced/spliced sequences. The resulting amino acid sequences were annotated using the online searching tool BLASTp at NCBI^80^. ORFs and spliced isoforms were named following the names described in the NCBI annotation with splicing sites noted as: I for intron and MI for multiple introns of a splicing event.

#### Comparison between related viral species

Multiple aligments were conducted for genome sequences using ClustalW^96^ to compare splicing junction locations between viral species. The graphical mapping details of final alignments were drawn using R.

#### Calculation of expression levels

A local estimation of each junctions were used to show their expression levels. The number of splicing and non-splicing reads located in junction regions were caculated based on the alignment Bam files. The number of non-splicing reads with donor or acceptor sites (reads spanning the exon-intron) were also caculated. We considered one ORF as one gene to calculate the expression levels. The expression level related values: i.e., the counts of positions of valid fragment (effective length), the sum of the posterior probability of each read coming from this part over all reads (expected count), the transcripts per million (TPM), and fragments per kilobase of transcript per million mapped reads (FPKM), were calculated using RSEM^70^ and Kallisto^71^. A relative expression level of each viral gene was measured using the FPKM value of one gene divided by the FPKM value of the highest expressed gene of the same virus.

### Validation of splicing products

#### Downsampling

1/10 and 1/20 subsamples of PcDNV related whole transcriptome reads were randomly selected from total reads using the toolkit Seqtk^97^ and analyzed using the same analytical pipeline conducted for the full transcriptomes to examine potential impact of sequencing depth.

#### Junction detection using STAR

STAR (version 2.5.3a) was applied to check the detected junctions using the parameters “–runMode genomeGenerate–sjdbOverhang 149–genomeChrBinNbits 12–genomeSAindexNbases 7 –sjdbGTFfile …” for STAR index construction and “–alignSJoverhangMin 5–alignIntronMin 50–alignIntronMax 2000–outSJfilterOverhangMin 5 5 5 5” for alignment. Then the alignment BAM file was used to define junctions using the script “Splicing_Search.pl” in pipeline after filtering with the same parameters as described in the TopHat2 step.

#### DNaseI treatment and reverse transcription

Total mRNAs were obtained from the 1KITE project directly and the quality was evaluated using a Qubit Fluorometer. To eliminate DNA contamination, 1 μg of total RNA was treated with 1 U DNaseI (Promega, US) and incubated at 37 °C for 30 min. Then samples (1 μg) of total RNA were reversely transcribed in a 25 μL reaction mixtures with oligo(dT)_15_ primers and M-MLV reverse transcriptase (Promega, US).

#### PCR

Splicing was validated by PCR of the cDNAs using primers designed from viral genome sequences from this study (Supplementary Table S8). The PCR mixture (50 μL) consisted of 4 μL cDNA template, 0.2 μM primers, 200 μM dNTP mix and 2.5 U TaKaRa LA Taq polymerase. The amplification conditions were denaturing at 94 °C for 5 min, then 40 cycles of denaturing at 94 °C for 30 s, annealing at 50 °C for 30 s, and extension at 72 °C (the extension time depends on the length of PCR amplicon, 1 kb/min), and final extension at 72 °C for 10 min.

#### Cloning and Sanger sequencing

PCR products were cloned into pEASY-T1 simple cloning vector (Transgen, China) and transformed into Trans1-T1 Phage resistant chemically competent cells (Transgen, China) for Sanger sequencing at Ruibiotech Company (Beijing, China).

### Data Availability

Raw data are available from NCBI bioprojects PRJNA286330 (*A*. *domesticus*) and PRJNA219593 (*P*. *citri*). The virus screening pipeline is available from https://github.com/linzhi2013/Virusfishing.

## Electronic supplementary material

Supplementary information

Supplementary Tables

Supplementary data files
