# Supplementary material for: Characterization of viral RNA splicing using whole-transcriptome datasets from host species
Source: Sci Rep. 2018 Feb 19;8:3273. doi: 10.1038/s41598-018-21190-7 (PMC5818608; doi:10.1038/s41598-018-21190-7)
Supplement: Supplementary file 1 — Supplementary information [file 41598_2018_21190_MOESM1_ESM.doc]

**Characterization of viral RNA splicing using whole-transcriptome datasets from host species**

Chengran Zhou^1, 2, 3^, Shanlin Liu^2, 3, 9^, Wenhui Song^2, 3^, Shiqi Luo^7^, Guanliang Meng^2, 3^, Chentao Yang^2, 3^, Hua Yang^1^, Jinmin Ma^2, 3^, Liang Wang^4^, Shan Gao^4^, Jian Wang^2, 5^, Huanming Yang^2, 5^, Yun Zhao^1, *^, Hui Wang^2, 3, 6, *^, Xin Zhou ^7, 8, *^

^1^ Key Laboratory of Bio-Resources and Eco-Environment of Ministry of Education, College of Life Sciences, Sichuan University, Chengdu 610065, China.

^2^ BGI-Shenzhen, Shenzhen 518083, China.

^3^ China National GeneBank, BGI-Shenzhen, Shenzhen 518120, China.

^4^ CAS Key Laboratory of Biomedical & Diagnostic Technology, CAS/Suzhou Institute of Biomedical Engineering and Technology, Suzhou 215163, China.

^5^ James D. Watson Institute of Genome Sciences, Hangzhou 310058, China

^6^ The Institute of Biomedical Engineering, University of Oxford, Oxford, OX3 7DQ, UK.

^7^ Beijing Advanced Innovation Center for Food Nutrition and Human Health, College of Plant Protection, China Agricultural University, Beijing 100193, China

^8^ National Engineering Research Center for Fruit and Vegetable Processing, China Agricultural University, Beijing 100193, China

^9^ Centre for GeoGenetics, Natural History Museum of Denmark, University of Copenhagen, Øster Voldgade 5-7, 1350 Copenhagen, Denmark

* Corresponding authors:

Xin Zhou, Email: xinzhou@cau.edu.cn

Hui Wang, Email: [huiwang789@gmail.com](mailto:huiwang789@gmail.com)

Yun Zhao, Email: zhaoyun@scu.edu.cn

**SUPPLEMENTARY TEXT S1**

**Phylogram of AdDNVs**

Phylogram based on AdDNVs genome sequences showed that the AdDNV_1KITE genome sequence was best matched to AdDNV_AdNA09 among all reported AdDNVs sequences available in the GenBank (Supplementary Fig. S2) ^1,2^.

**Expressions of conserved motifs**

As in parvoviruses, several conserved protein domains play important roles ^3-5^. A conserved sequence domain located in NS proteins known as Parvo_NS1 appeared to be highly expressed in both AdDNV_1KITE (translated region from nt 2,119 to 2,433, Fig. 2C) and PcDNV_1KITE (nt 2,034 to 2,381, Fig. 2D). This region is known as a highly conserved protein domain - parvovirus non-structural protein NS1, which is essential for DNA replication in parvovirus ^6^. Another conserved protein motif (Pavo_coat_N) ^7^ was also highly expressed in both AdDNV_1KITE (nt 4,750 to 4,613) and PcDNV_1KITE (nt 4,748 to 4,650) (Fig. 2). A region of capsid protein VP4 (Denso_VP4) was translated from nt 3,882 to 2,674 in AdDNV_1KITE and from nt 3,952 to 2,672 in PcDNV_1KITE, respectively. The region of phospholipase A2 (PLA2) motif, located in capsid proteins, which were critical for efficient viral transfection and infection ^7,8^ , also showed high levels of expression between nt 4,684 to 4,649 in AdDNV_1KITE and nt 4,682 to 4,647 in PcDNV_1KITE, respectively (Fig. 2). The unevenness of coverage (Fig. 2) may suggest possibilities of novel transcripts.

**Gene products of AdDNV**

In the NS coding region, Figure 4A showed the locations of four NS ORFs. As previously reported ^1^, AdDNV_I1 was a major splicing event which eliminate the coding region of AdDNV_NS_ORF1 (NS3), resulted in productions of NS1 (AdDNV_NS_ORF2) and NS2 (AdDNV_NS_ORF3). A minor NS3 isoform (AdDNV_NS_ORF1_I2) was detected as a 68 aa long peptide containing the N-terminus of NS3 (Fig. 4A).

In the AdDNV_VP region, also as previously reported ^1^, a dominant splicing event of AdDNV_VP_ORF6_I4 combined two original ORFs (AdDNV_VP_ORF4 and AdDNV_VP_ORF5, Fig. 4B) to produce an 816 aa long protein (AdDNV_VP1). A minor splicing event (AdDNV_VP_ORF6_I3) occurred at a different junction produced a 38 aa truncated isoform of the VP1 (Fig. 4B). Another shorter A3SS splicing event (AdDNV_VP_ORF5_I5) produced a 233 aa long protein, a C-terminal truncated form of the original 265 aa VP_ORF5 (Fig. 4B).

**Gene products of PcDNV**

PcDNV displayed a set of seven A5SS introns with a shared receptor junction at nt 879. Although the locations of these A5SS introns were comparable to the AdDNV NS introns (Fig. 3E), their donor junction occurred downstream of the PcDNV_NS_ORF1 (homologue of AdDNV_NS3) start codon and the receptor junction located downstream of the start codon of PcDNV_NS_ORF2 (homologue of AdDNV_NS1) at the start codon position of PcDNV_NS_ORF3 (homologue of AdDNV_NS2). With different donor positions, the PcDNV NS introns produced a set of NS proteins (Fig. 4C). The dominant NS splicing was PcDNV_NS_ORF7_I2, resulted in a substitution of the PcDNV_NS_ORF2 N-terminal 23 aa by the 20 aa N-terminal of the PcDNV_NS_ORF1. Four additional isoforms of the ORF1-ORF2 protein were produced by minor splicing events resulting in increased ORF1 proportions at the N-termini (Fig. 4C). The second dominant PcDNV NS splicing (PcDNV_NS_ORF6_I1) resulted in a full length PcDNV_NS_ORF3 (homologue of AdDNV_NS2) with an additional ORF1 section (19 aa long) at the N-terminus. An isoform of this ORF1-ORF3 with the N-terminal ORF1 part extended to 48 aa was produced by the third most popular intron PcDNV_NS_ORF6_I4 (Fig. 4C).

*PcDNV_VP_ORF8.* The most popularly detected VP splicing in PcDNV was PcDNV_VP_ORF8_I16 with 1,994 supporting reads (Fig. 4D). PcDNV_VP_ORF8_I16 eliminated the stop codon of PcDNV_VP_ORF5 and joined the PcDNV_VP_ORF4 (homologue of AdDNV-VP2) reading frame, producing an 838 aa long protein (homologue of AdDNV-VP1). Two more 3ASS splicing events were detected for this donor junction.

*PcDNV_VP_ORF9.* The other one 3ASS splicing event (PcDNV_VP_ORF9_I18, was supported with one supporting read) produced isoform of the PcDNV_VP_ORF5 protein for which the C-termini was replaced by a novel sequence of 34 aa long fraction. BLASTp search (E value < 1) against the NCBI nr database for this novel peptide did not yield any homologue.

*PcDNV_VP_ORF10.* The 2^nd^ most popular PcDNV_VP intron was PcDNV_VP_ORF10_I23 supported with 1,699 reads. It had a minor 5ASS isoform (PcDNV_VP_ORF10_I22) and both produced the N-terminal fractions of the PcDNV_VP_ORF5 protein followed by a 96 aa long novel peptide at the C-termini (Fig. 4D). Again, no homologue was detected from the NCBI by the BLASTp search for this novel peptide sequence. It was worth to note that the conserved Parvo_coat_N region and PLA2 motif were deleted by these PcDNV_VP_ORF10 splicing events.

*PcDNV_VP_ORF5.* The 3^rd^ most popular PcDNV_VP splicing event produced PcDNV_VP_ORF5_I20 (432 supporting reads) which was a C-terminal 16 aa truncated isoform of the PcDNV_VP_ORF5 protein. Its 5’-donor junction had three A3SS isoforms, PcDNV_VP_ORF5_MI1, PcDNV_VP_ORF8_I14 and _I17. The former produced a truncated isoform of the PcDNV_VP_ORF5 protein and the latter two produced isoforms of the PcDNV_VP1 (PcDNV_VP_ORF8) protein (Fig. 4D).

*PcDNV_VP_ORF4.* The 4^th^ most popularly detected A3SS splicing pair was PcDNV_VP_ORF4_I12 (291 supporting reads) and _I13 (44 supporting reads), both produced small proteins of 176-179 aa long containing the N-terminal part of PcDNV_VP_ORF4 but without the conserved Denso_VP4 motif (Fig. 4D). In addition to these two introns, five more splicing events were detected in the PcDNV_VP_ORF4 region. A rare splicing event produced a 34 aa long peptide (PcDNV_VP_ORF4_I15 with a single supporting read) while the others produced truncated versions (561-599 aa long) the PcDNV_VP_ORF4 (homologue of AdDNV_VP2) (Fig. 4).

**Gene expression characteristics**

In AdDNV, AdDNV_NS_ORF2 (Fig. 4A) had the highest FPKM value of 328,484.48 indicating that the encoded AdDNV_NS1 was the most abundantly expressed protein (Table 2). NS_ORF1 (encoding AdDNV_NS3) had a FRKM value of 18,362.89, only 5.59% of NS_ORF2 (AdDNV_NS1), but it was much higher than that of AdDNV_NS_ORF3 (encoding AdDNV_NS2) (Fig. 4A, Table 2). In the VP region, AdDNV_VP_ORF6_I4 had a FPKM value of 164,855.44 and was the highest expressed VP isoform (VP1), showing the importance of splicing event AdDNV_I4 (Fig. 4B). The relative expression level of AdDNV_VP1 was notably less (68% reduction) than that of AdDNV_NS1 (Table 2).

In PcDNV, PcDNV_NS_ORF7_I2 (FPKM value 182,580.84, AdDNV_NS1 homologue) was the most abundantly expressed PcDNV_NS protein, followed by PcDNV_NS_ORF6_I1 (FPKM value 18,452.28, AdDNV NS2 homologue), PcDNV_NS_ORF1 (FPKM 17,344.87, AdDNV_NS3 homologue) and PcDNV_NS_ORF2 (FPKM 15,339.25, AdDNV_NS1 homologue) (Fig. 4C, Table 2). PcDNV_VP_ORF10_I23 (FPKM value 269,981.32), which encoded a novel protein without any conserved *Ambiensovirus* VP motifs ^1,9,10^, was the most abundantly expressed PcDNV_VP protein (Fig. 4D, Table 2), followed by PcDNV_VP_ORF8_I16 with FPKM value 185,726.27 and PcDNV_VP_ORF4 with FPKM value 107,736.5.

**Differences among AdDNV and PcDNV structural and non-structural proteins**

Splicing regulation is more complex for VP mRNAs than for NS mRNAs. In PcDNV, a novel small protein (PcDNV_VP_ORF10_I23, 185 aa, Fig. 4D) was produced as the most abundant protein. The deduced amino acid sequence of its C-terminal half did not resemble any known protein in the public databases, highlighting a knowledge gap in the understanding of *Ambidensovirus*. For the non-structural proteins, although both viruses produced their most abundant NS isoforms (AdDNV NS1) via AS, different translation strategies were observed. AdDNV_I1 skipped the AdDNV_NS1 start codon and translation started downstream of the AdDNV_NS1 coding region (Fig. 4A) ^1^. Its homologue PcDNV_NS_ORF7_I2, however, had a 20 aa PcDNV_NS_ORF1 (AdDNV_NS3 homologue) N-terminus followed by the truncated PcDNV_NS_ORF2 (544 aa long, AdDNV_NS1 homologue) (Fig. 4C). Such a difference may suggest N-terminal mediated functional divergence and/or difference on the efficiency of NS start codons.

**Virus-host association**

From the virology point of view, it would be reasonable to expect that two closely related viruses, AdDNV and PcDNV (Supplementary Fig. S1), should share a similar gene expression strategy in their natural hosts ^11^. Indeed, the genome architectures are similar between the two viruses ^1,12^. And in this study, a conserved AS rule was detected for producing the AdDNV_VP1 (AdDNV_VP_ORF6_I4, Fig. 4B) and its counterpart in PcDNV (PcDNV_VP_ORF8_I16, Fig. 4D). Although the most abundantly expressed NS isoforms in AdDNV (NS1, AdDNV_NS_ORF2, Fig. 4A) and PcDNV (PcDNV_NS_ORF7_I2, Fig. 4C) were largely similar as the AdDNV_NS1 analogue, the highest FPKM value of PcDNV_VP products was a completely novel protein (PcDNV_VP_ORF10_I23, Fig. 4D and Table 2) which C-terminal half did not resemble to any of the known protein in the public database. The high expression level strongly suggested important function(s) of this novel protein. Because RNA splicing mechanism in viruses needs to involve virus factors and host machinery in a host-specific manner ^13^, it is plausible that PcDNV produces additional viral protein(s) to support the complication of its AS activities (Fig. 3E).

**REFERENCES**

1 Liu, K. *et al.* The Acheta domesticus densovirus, isolated from the European house cricket, has evolved an expression strategy unique among parvoviruses. *J Virol* **85**, 10069-10078, doi:10.1128/JVI.00625-11 (2011).

2 Pham, H. T. *et al.* Comparative genomic analysis of Acheta domesticus densovirus isolates from different outbreaks in Europe, North America, and Japan. *Genome announcements* **1**, e00629-00613 (2013).

3 Cotmore, S. F. & Tattersall, P. Parvovirus DNA Replication. *Cold Spring Harbor Monograph Archive* **31**, 799-813 (1996).

4 Finn, R. D. *et al.* Pfam: the protein families database. *Nucleic Acids Res* **42**, D222-230, doi:10.1093/nar/gkt1223 (2014).

5 Marchler-Bauer, A. *et al.* CDD: NCBI's conserved domain database. *Nucleic Acids Res* **43**, D222-226, doi:10.1093/nar/gku1221 (2015).

6 Nüesch, J. P. & Tattersall, P. Nuclear targeting of the parvoviral replicator molecule NS1: evidence for self-association prior to nuclear transport. *Virology* **196**, 637-651 (1993).

7 Tullis, G. E., Burger, L. R. & Pintel, D. J. The minor capsid protein VP1 of the autonomous parvovirus minute virus of mice is dispensable for encapsidation of progeny single-stranded DNA but is required for infectivity. *Journal of virology* **67**, 131-141 (1993).

8 Zádori, Z. *et al.* A viral phospholipase A 2 is required for parvovirus infectivity. *Developmental cell* **1**, 291-302 (2001).

9 Tijssen, P. *et al.* Organization and Expression Strategy of the Ambisense Genome of Densonucleosis Virus of Galleria mellonella. *Journal of Virology* **77**, 10357-10365, doi:10.1128/jvi.77.19.10357-10365.2003 (2003).

10 Ward, A. J. & Cooper, T. A. The pathobiology of splicing. *J Pathol* **220**, 152-163, doi:10.1002/path.2649 (2010).

11 Yu, Q. & Tijssen, P. Gene expression of five different iteradensoviruses: Bombyx mori densovirus, Casphalia extranea densovirus, Papilio polyxenes densovirus, Sibine fusca densovirus, and Danaus plexippus densovirus. *J Virol* **88**, 12152-12157, doi:10.1128/JVI.01719-14 (2014).

12 Thao, M. L., Wineriter, S., Buckingham, G. & Baumann, P. Genetic characterization of a putative Densovirus from the mealybug Planococcus citri. *Curr Microbiol* **43**, 457-458, doi:10.1007/s002840010339 (2001).

13 Cullen, B. R. The virology-RNA biology connection. *RNA* **21**, 592-594, doi:10.1261/rna.049882.115 (2015).

**SUPPLEMENTARY CAPTIONS AND LEGENDS**

Supplementary Text S1.

Supplementary Table S1. Putative viral sequences details.

Supplementary Table S2. Statistics of reference-based genome reassembly.

Supplementary Table S3. RNA-seq mapping details.

Supplementary Table S4. More details on detected introns.

Supplementary Table S5. Splice junctions with multiple introns of PcDNV_1KITE.

Supplementary Table S6. Viral gene products and their expression levels.

Supplementary Table S7. RNA information.

Supplementary Table S8. Primer sequences.

Supplementary Figure S1. Phylogenetic trees of densoviruses. (A). Maximum likelihood (ML) tree based on genome sequences. (B). ML tree based on non-structural protein sequences. (C). Neighbour joining (NJ) tree based on genome sequences. (D). NJ tree based on non-structural protein sequences. Phylograms were built with the assembled AdDNV_1KITE and PcDNV_1KITE sequences together with DNV sequences downloaded from the NCBI, including the *P. citri* densovirus (PcDNV, NC004289.1), *A. domesticus* densovirus (AdDNV AdNA09, KF015278.1), *Culex pipiens* densovirus (CpDNV, NC012685.1), *Galleria mellonella* densovirus (GmDNV, NC004286.1), *Blattella germanica* densovirus (BgDNV, NC005041.2), *Periplaneta fuliginosa* densovirus (PfDNV, NC000936.1), *Diatraea saccharalis* densovirus (DsDNV, NC001899.1), *Bombyx mori* densovirus 5 (BmDNV-5, NC004287.1), *Casphalia extranea* densovirus (CeDNV, NC004288.1), *Aedes albopictus* densovirus 2 (AalDNV-2, NC004285.1), and *Penaeus monodo*n hepandensovirus1 (PmHPV-1, NC007218.1). The bootstrap values were labeled next to the branches. The trees were drawn to scale, with branch lengths measured in the number of substitutions per site.

Supplementary Figure S2. Phylogenetic tree of AdDNVs.

Supplementary Figure S3. Logo of PcDNV junctions with coverage depth greater than 2.

Supplementary Figure S4. RT-PCR analysis of PcDNV and AdDNV.

Supplementary Data file S1. BWA alignment profiles of AdDNV and PcDNV.

Supplementary Data file S2. Tophat2 alignment profiles of AdDNV and PcDNV

Supplementary Data file S3. High identities result from protein examination step.

**SUPPLEMENTARY FIGURES**

Supplementary Figure S1. Phylogenetic trees of densoviruses.

Supplementary Figure S2. Phylogenetic tree of AdDNVs.

Supplementary Figure S3. Logo of PcDNV junctions with coverage depth greater than 2.

**
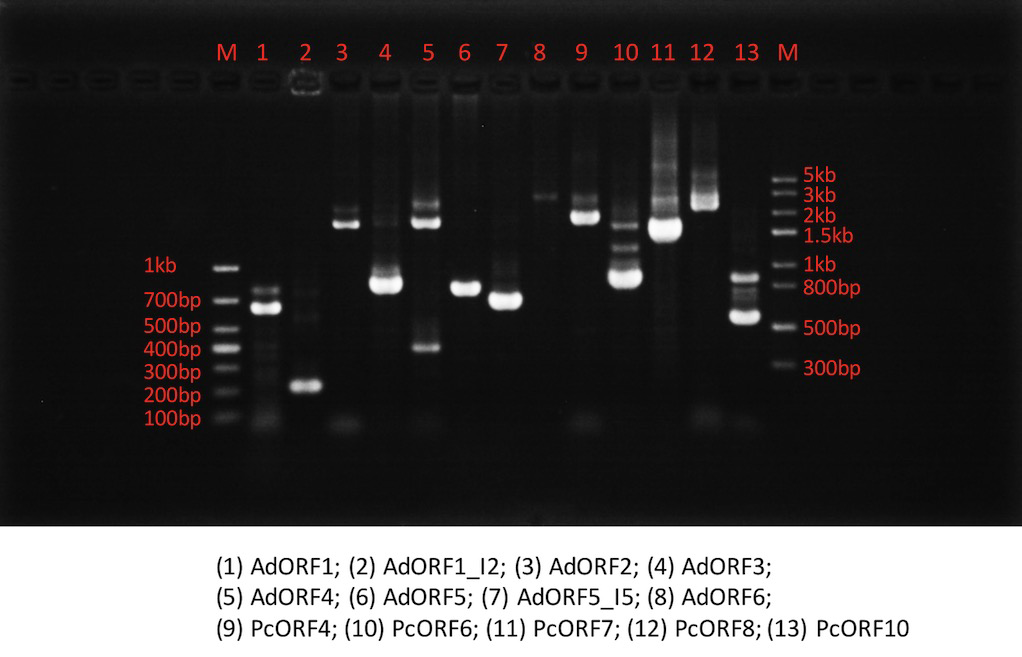
**

Supplementary Figure S4. RT-PCR analysis of PcDNV and AdDNV.
